# Intraoperative contrast-enhanced ultrasonography for microcirculatory evaluation in rhesus monkey with spinal cord injury

**DOI:** 10.18632/oncotarget.17252

**Published:** 2017-04-19

**Authors:** Lin Huang, Keng Chen, Fu-Chao Chen, Hui-Yong Shen, Ji-Chao Ye, Zhao-Peng Cai, Xi Lin

**Affiliations:** ^1^ Department of Orthopedics, Memorial Hospital of Sun Yat-Sen University, Institute of Spinal Cord Injury, Sun Yat-Sen University, Guangzhou, Guangdong Province, 510120, P.R. China; ^2^ Department of Ultrasound, Sun Yat-Sen University Cancer Center, State Key Laboratory of Oncology in South China, Guangzhou, Guangdong Province, 510060, P.R. China

**Keywords:** spinal cord, contusion, perfusion, rhesus monkey, contrast-enhanced ultrasound

## Abstract

This study tried to quantify spinal cord perfusion by using contrast-enhanced ultrasound (CEUS) in rhesus monkey models with acute spinal cord injury. Acute spinal cord perfusion after injury was detected by CEUS, coupling with conventional ultrasound (US) and Color Doppler US (CDFI). Time-intensity curves and perfusion parameters were obtained by autotracking contrast quantification (ACQ) software in the epicenter and adjacent regions of injury, respectively. Neurological and histological examinations were performed to confirm the severity of injury. US revealed spinal cords were hypoechoic and homogeneous, whereas dura maters, pia maters, and cerebral aqueducts were hyperechoic. After spinal cord contusion, the injured spinal cord was hyperechoic on US, and intramedullary vessels of adjacent region of injury were increased and dilated on CDFI. On CEUS hypoperfusion were found in the epicenter of injury, while hyperperfusion in its adjacent region. Quantitative analysis showed that peak intensity (PI) decreased in epicenters of injury but significantly increased in adjacent regions at all time points (*p* < 0.05). Functional evaluation demonstrated significant deterioration compared to pre-contusion (*p* < 0.05). Quantitative analysis with CEUS is a promising method for monitoring perfusion changes of spinal cord injury in overall views and real-time.

## INTRODUCTION

Vascular events have critical effects on spinal cord in primary and secondary injury. The initially mechanical insults primarily cause vessel disruption and damage blood supply of the spinal cord. Subsequently, tissue edema or hemorrhage and ischemia play important roles in expansion of injury. Therefore, quantitative measurement of spinal cord blood flow (SCBF) is valuable for understanding pathophysiologic mechanisms of spinal cord injury (SCI) [1, 2]. Previously, progressive “posttraumatic ischemia” has been recorded in a number of studies by using various methods for SCBF [1–4]. These techniques include hydrogen clearance, radioactive tracer microsphere and laser Doppler flowmetry. However, few studies show perfusion changes in parenchyma of contusive spinal cord in overall views and real-time [5].

Contrast-enhanced ultrasound (CEUS) offers major advantages of assisting in evaluation of spinal cord perfusion in real-time, without any risk of ionizing radiation [4, 6] compared with other methods, which were used to assess blood flow. Recently, CEUS has been tested in assessment of cerebral perfusion deficits in stroke patients [7].

As to animal model of SCI, numerous experimental studies have been conducted using rodents. However, there are differences between rodents and human beings in the neurofunctional and anatomic features. Therefore the results cannot be applied directly to patients with SCI. Nonhuman primate models have contributed greatly to advance research in nervous system diseases such as parkinsonism, multiple sclerosis hemisection and graded SCI [8].

The purpose of this study was to quantify perfusion characteristics of spinal cord by using CEUS in rhesus monkey models with spinal cord contusion.

## RESULTS

### Characteristics of ultrasound imaging and quantitative analysis

Hypoechoic and homogeneous echo pattern was found in parenchyma of all intact spinal cords (Figure 2A). Dura mater and cerebral aqueduct appeared as hyperechoic on conventional ultrasound. By using CDFI, we were able to observe segmental and columnar intramedullary vessels in a sagittal plane (Figure 2B).

**Figure 1:**
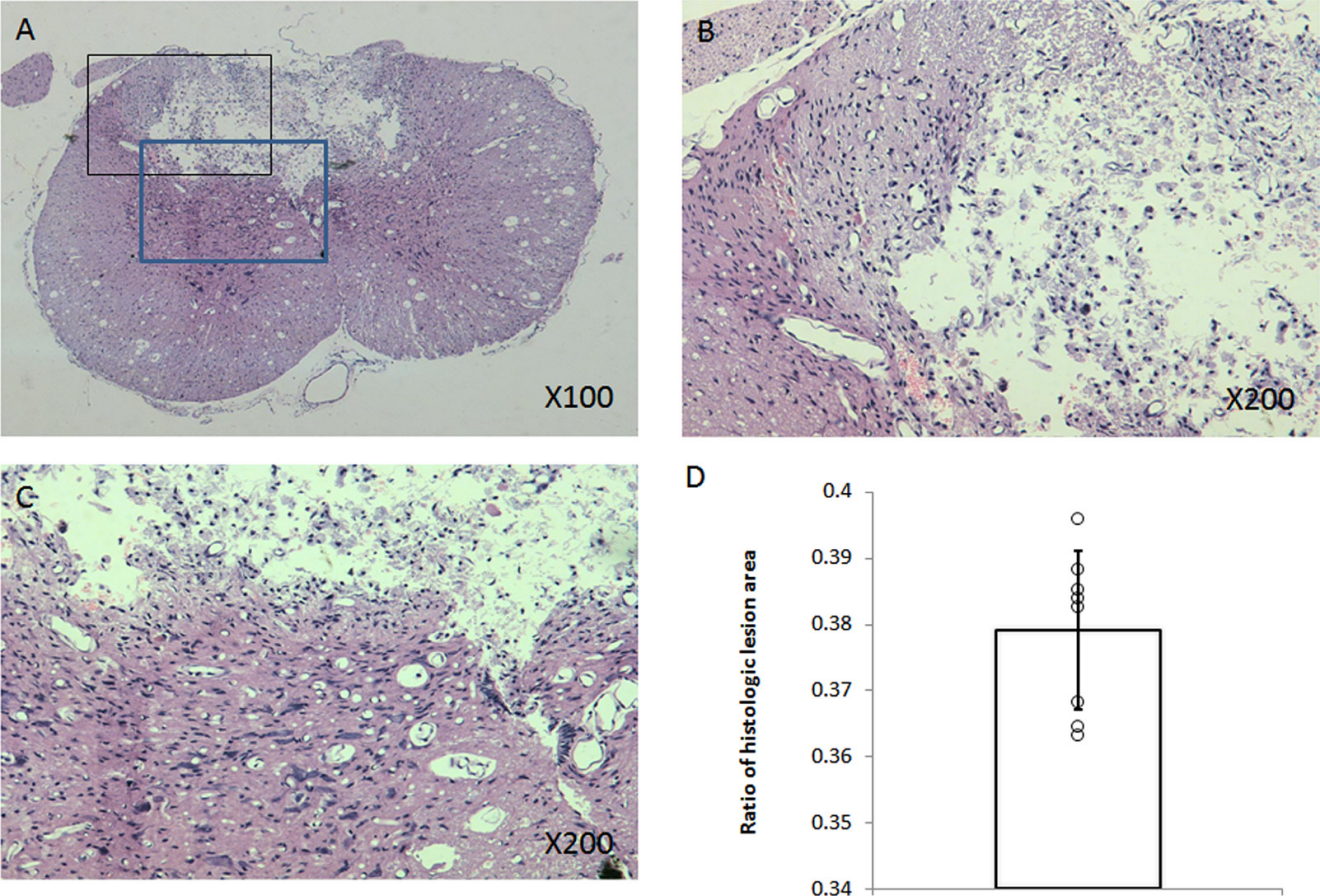
Histopathologic examination Representative H&E stained section showed that cavity lesion in the contusion epicenter was surrounded by glia scar and demyelination in near grey matter or white matter (**A**, **B**, **C**). Diagram demonstrated ratio of histologic lesion area (**D**).

**Figure 2:**
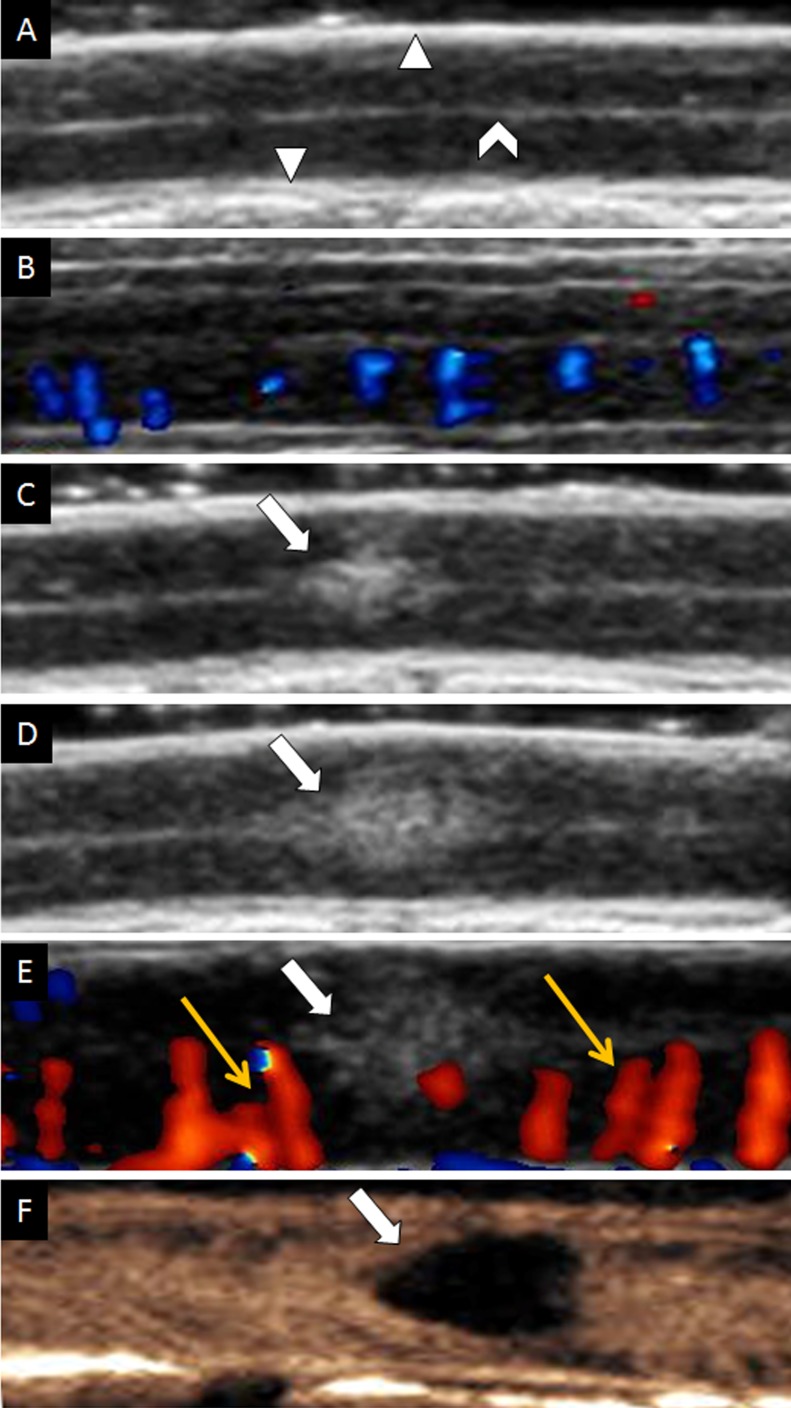
Ultrasound imaging of spinal cord on longitudinal view (**A**) Gray scale of uninjured spinal cord. (**B**) CDFI of uninjured spinal cord. (**C**) Contusive spinal cord instantly on gray-scale sonography. (**D**) Increasing size of contusive spinal cord on gray-scale sonography. (**E**) CDFI of injured spinal cord. Yellow arrows indicated dilated vessels. (**F**) CEUS showed the contusive lesion was non-perfusion. The white triangle indicated dura mater of spinal cord; The white caret indicated cerebral aqueduct. The white arrow indicated contusive lesion.

However, injured region of spinal cord parenchyma was appeared as hyperechoic and its area increased gradually over time (Figure 2C, 2D) on conventional ultrasound. In the same time, we demonstrated that intramedullary blood flow dramatically increased and vessels obviously dilated in regions close to injury immediately after contusion (Figure 2E) by CDFI. The average diameter of vessels in the adjacent regions of injury before contusion was 0.465 ± 0.054 mm (range, 0.38 to 0.53 mm) and it was increased by about 0.15 mm immediately after contusion (0.611 ± 0.076 mm, range, 0.49 to 0.72 mm) (*p* < 0.01).

**Figure 3:**
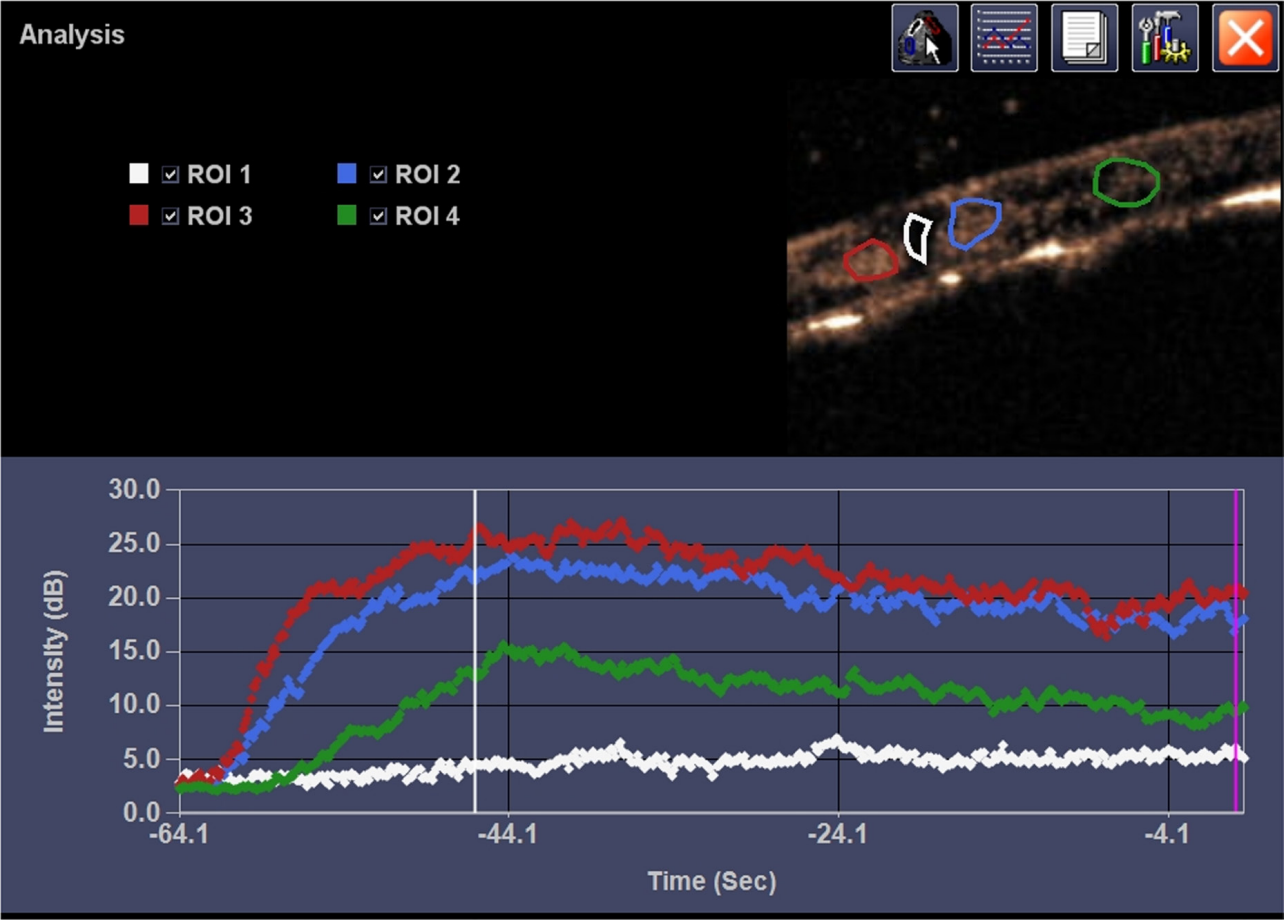
Representative CEUS and ACQ curves It showed the pattern of ischemic region in contused site after SonoVue infusion and enhancement in adjacent region. ROI 1, ROI 2, ROI 3 and ROI 4 represented the epicenter of contusion site, adjacent region caudal, adjacent region rostral, and distant region, respectively.

**Figure 4:**
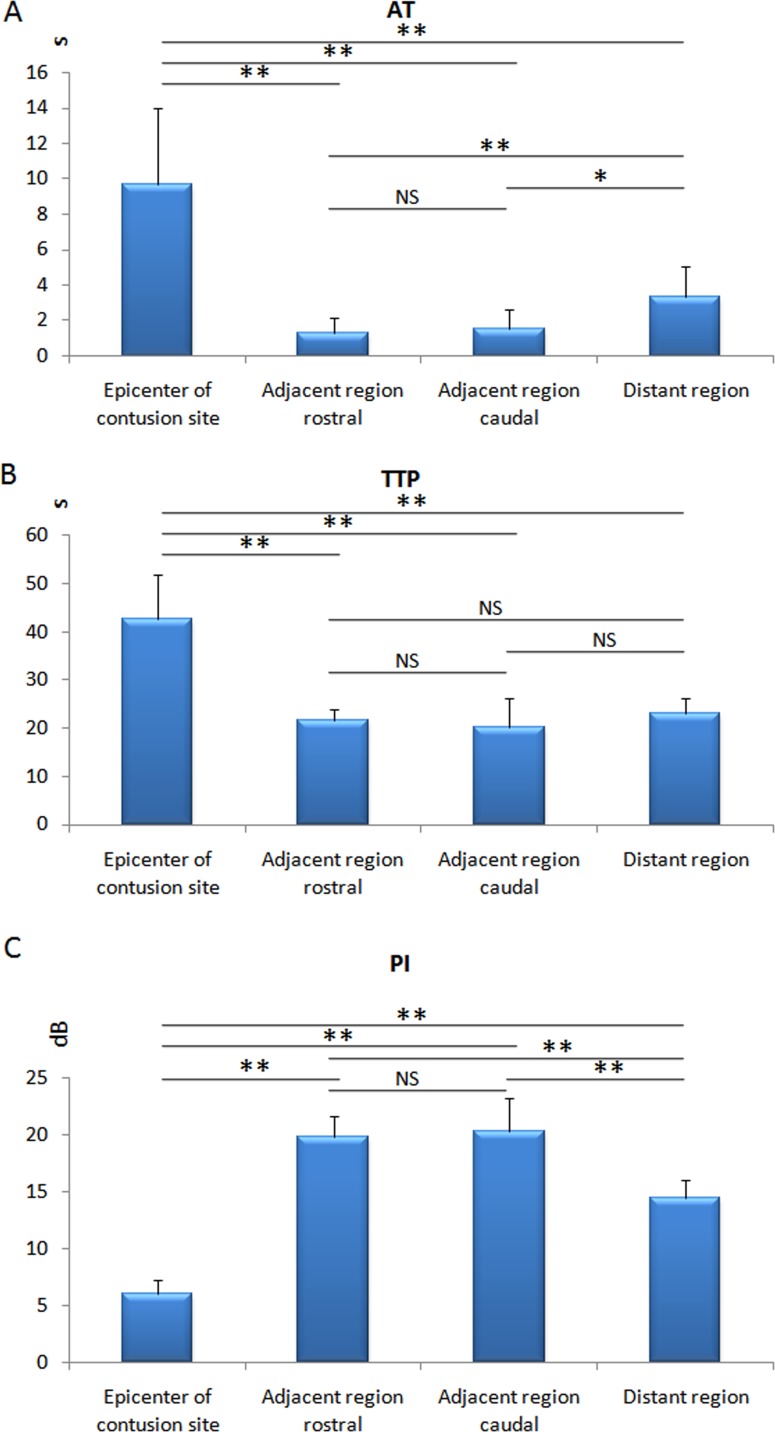
Diagram of the AT, TTP and PI Significantly prolonged in AT and TTP of epicenter of contusion and shortened in AT of adjacent region (rostral and caudal), as compared to distant region; PI decreased in the epicenter of contusion but increased in the adjacent region (rostral and caudal) significantly. *p < 0.05, **p < 0.01; NS: no statistical significant.

On CEUS, blood perfusion of intact spinal cord was homogeneous and became heterogeneous after contusion (Figure 2F). Fragmentary rim-like enhancement was detected around the epicenter of contusion by CEUS. We found hypoperfusion in injured site and hyperperfusion in its adjacent region (rostral and caudal) at the same time. Quantitative analysis by ACQ software showed AT and TTP were significantly prolonged in the epicenter of injury (*p* < 0.05), whereas they were shortened in adjacent region of injury (*p* < 0.05) (Figure 3). Unlike AT and TTP, PI was dramatically decreased in epicenter of injury but remarkably increased in adjacent region (rostral and caudal) of injury (*p* < 0.05) (Figure 4).

### Neurological deficit and tissue damage

**Figure 5:**
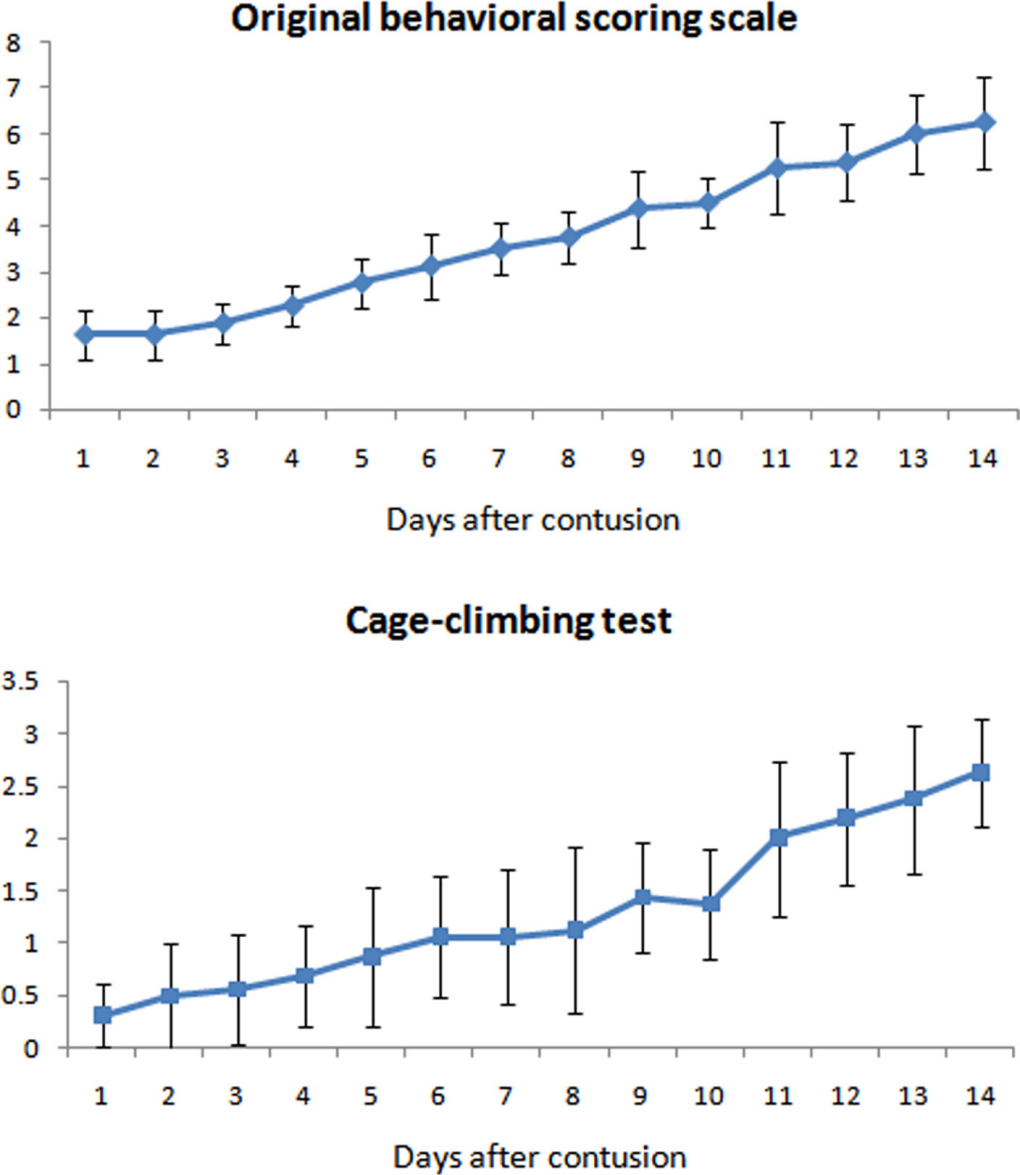
Neurological function of the animals evaluated by two methods: Original behavioral scoring scale and cage climbing test It showed that neurologic status recovered gradually in 14 days.

Neurological function was immediately deteriorated after contusion and gradually recovered over time (Figure 5). The mean original behavioral scoring scale was approximately 2 on Day 1st after injury and increased gradually to approximately 6 on Day 14th after injury. Mean cage-climbing test score decreased to 0.31 ± 0.30 on Day 1st after injury and increased to 2.63 ± 0.52 on Day 14th after injury.

Histological analysis showed that cavity lesion in the contusion epicenter was surrounded by glia scar. Demyelination was also found in adjacent regions of injury (Figure 1). A negative correlation between ratio of histologic lesion area and original behavioral scoring or cage-climbing test on Day 14th after injury was found (*r* = −0.58, *r* = −0.76, respectively).

## DISCUSSION

More and more studies confirm that the secondary SCI is crucial to the degree of neurological deficit of SCI patients. Perfusion plays a critical role in both primary and secondary spinal cord injury [2, 9, 10]. Perfusion monitoring lays the foundation in exploring mechanisms of secondary SCI. There are several techniques for monitoring spinal cord perfusion, including hydrogen clearance method, radioactive tracer microsphere technique or Laser Doppler Flowmetry technique, magnetic resonance imaging (MRI) and positron emission tomography (PET), computed tomography (CT). However, there are some disadvantages that should be considered. Laser Doppler was a popular, conventional and real time technique for SCBF in previous studies. However, its limited detecting ability was only up to a 3 mm depth of spinal cord [5]. This made it difficult to monitor SCBF in deep spinal cord tissue in large animals. Therefore, it could not provide an overall view of spinal cord blood perfusion. Some studies have found it too sensitive in artifacts [4, 11, 12]. The use of imaging modalities such as MRI in detecting SCBF is being investigated. Recently, the first report has been presented on quantitative SCBF measurements in mice by MRI with a pulsed arterial spin labeling (ASL) technique based on a presaturated flow-sensitive alternating inversion recovery (presat-FAIR) EPI sequence [13]. ASL is a typical non-invasive MRI approach to measure perfusion. Its basic principle is that blood is magnetically labeled and serves as an intrinsic contrast agent. However, it is not a continuous measurement of tissue perfusion. And FAIR is a technique with low sensitivity in human studies. Although enhanced CT and MRI are also used to detect perfusion patterns in clinical, it is inconvenient for intraoperative monitoring of spinal cord perfusion. Actually, clinical application of MRI in spinal cord perfusion is limited for both resolution and small size of the cord. In experimental study, furthermore, an expensive and special coil is needed for animals.

SonoVue is a real blood pool imaging agent which does not diffuse through the vascular endothelium into interstitium and without risk of ionizing radiation. As a result, contrast enhancements represent tissue vascularity. It is available to maximally preserve microbubbles in circulation with low-MI contrast-enhanced sonography, leading to a continuous real-time scanning mode. Therefore, arrival time of the contrast agent in the target region and its fading out can be dynamically observed, which facilitates assessment of the vascularity of spinal cord. With objective methods such as time-intensity curves, CEUS depicts the enhancement with time. Moreover, CEUS is easy to perform on spinal cord when it is exposed during operation. Therefore, in comparison to those methods mentioned above, CEUS has some advantages, including better availability, subject comfort, and no exclusion of patients or animals with internal metal fixation. What's more, CEUS enable the simultaneous measurement of tissue perfusion in real time and is convenient for bedside measurement e.g., for intraoperative animals or patients. The results of this study demonstrated that quantitative analysis with CEUS was a practical and useful method for assessment of spinal cord perfusion in real-time and overall views.

In our spinal cord contusion models, a corresponding increase in size of hyperechoicity in contusive epicenter was observed on conventional US, which had less blood flow on CDFI. This result was consistent with what was previously reported [14]. Possible explanation for hypofusion in contusion site was that the initial impact disrupted blood supplies within the spinal cord and resulted in local infarction caused by hypoxia and ischemia [10]. Moreover, hypoperfusion frequently indicated vascular damage from immediate vasospasm of superficial vessels to intraparenchymal hemorrhage and the formation of thrombosis [10, 15, 16]. The intact perfusion of distant region served as a good reference.

As to adjacent region to the contusion site, significant posttraumatic hypoperfusion in adjacent segments of spinal cord has been reported in literatures [17–20]. Likewise, a majority of clinical and experimental studies have shown less cerebral blood flow in and around contused brain parenchyma [15, 21, 22]. However, the most significant difference in our study from previous studies was in the perfusion in adjacent regions. There were more dilated vessels in surrounding region than the contusion site. Using CEUS, hyperechoic enhancement pattern was observed and the PI of adjacent regions either rostral or caudal was significantly higher than that of epicenter and distant regions by quantitative analysis (*P* < 0.05).

Dilated microvessels, failed autoregulation, or opening arteriovenous shunt underlied hyperperfusion in adjacent region. Our study demonstrated that significant vessels dilation in adjacent region on CDFI. It was similar to the report by Chen [23]. They found average diameters of cortical microvessels were significantly increased in the contusion margin at the initial time points in a moderate controlled cortical impact injury model. Vessel dilation in adjacent region was a response to the mechanical insult and metabolic demand. The latter was due to acute increasing in oxygen consumption and glucose metabolism after trauma [24]. The findings by Chen [23] also indicated that microvascular density (MVD) was significantly reduced in the contusion margin. In contrast, our data showed that there was a correspondingly significant hypervascularity and hyperperfusion in the adjacent region by CDFI and quantitative analysis with CEUS. This finding suggested that spinal cord perfusion determined by CEUS could be more reliable than MVD measurements. Our result was reasonable because CEUS could be used to assess much larger volumes of spinal cord than volumes of tissue used for histological analysis. Additionally, MVD cannot provide information on effective perfusion through blood vessels.

The hemodynamic changes were vividly shown with CEUS in real-time. CEUS thus could be employed in other type of experimental SCI, such as compression, chronic SCI and even in clinical applications, which includes traumatic, iatrogenic SCI or spinal cord tumors during operation. Potential clinical application of contrast agents was shown in not only diagnostic but also therapeutic aspects [25]. Using CEUS combined with neuroprotective or therapeutic genes or drugs could be a new strategy for SCI.

The limitations in our study were limited time course and no corresponding immediate histologic examination. There was lack of graded contusion models with different impact energy. Further studies are needed in evaluating the role of enhancement of adjacent region in secondary injury and in investigating its underlying mechanisms.

## MATERIALS AND METHODS

### Contusive SCI in rhesus monkeys

Experimental protocols were approved by animal care and use committee of our hospital under the Guild for the Care and Use of Laboratory Animals. Eight adult rhesus monkeys of 4 years old (5 males), weighing from 9 to 11 kg, underwent contusive SCI with a modified spinal cord impactor system.

**Figure 6:**
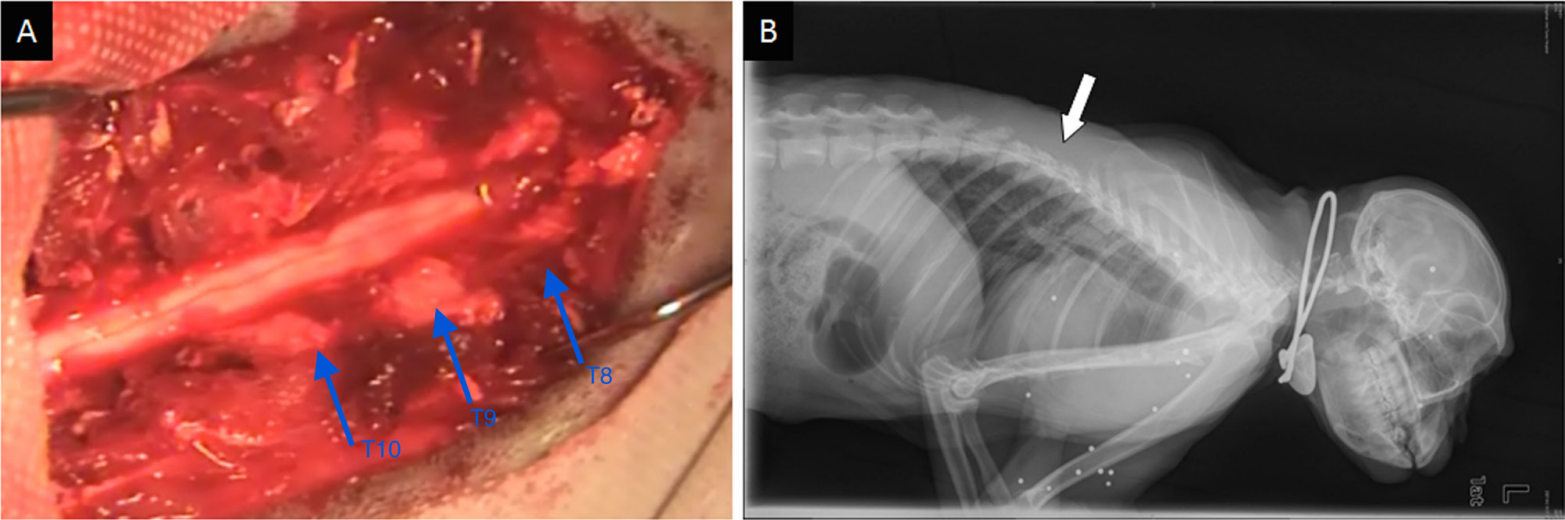
Intraoperative photographs and X-ray plain of the rhesus monkey model (**A**) Exposure of the spinal cord from T8–T10. (**B**) X-ray confirmed the operated segments. White arrow showed the field of operation.

After an intramuscular injection of 50 mg/kg ketamine and 5 mg/kg xylazine to induce anesthesia, animals were further anesthetized with slowly administration of intravenous Thiamylal (1.5 mg/kg). Then an exposure of thoracic cord with intact dura mater was achieved by total laminectomy from T8 to T10 (Figure 6). Contusive SCI was induced with a 50-g weight dropped from a height of 15 cm. Ultrasound scanning protocol was employed before and after injury. Animals were placed in a separate cage in a temperature-controlled chamber until thermoregulation was recovered. Food and water were fed with assistance until their trophic abilities recovered. Manual bladder expression was carried out twice a day until voiding reflexes were reestablished, paracentesis of bladder was used in necessary. Buprenorphine (0.1 mg/kg) and Ceftiofur (4 mg/kg) were intramuscularly administered for 5 days after injury as an analgesic and an antibiotic, respectively.

### Scanning procedures

Before contusive injury, the spinal cord was imaged with gray-scale ultrasound, color Doppler flow imaging (CDFI), and CEUS. Then, contusive SCI was performed by using the modified spinal impactor device. Ultrasound scanning procedures were performed again after the impactor was removed from the contusive spinal cord immediately. The CDFI images provided the diameters of intramedullary blood vessels on the same section pre- or post-contusion. CEUS was performed with a linear transducer 15L8w (Acuson Sequoia 512, Siemens, Mountain View, Calif, USA), which is equipped with contrast-specific, real-time imaging technology (contrast pulse sequencing). SonoVue^®^ (Bracco, Milan, Italy) was prepared just before administration by adding 6.0 mL of sterile saline solution (0.9% NaCl) to the vial and then vigorously shaking it for at least 20 s. The transducer was kept as still as possible on the previously selected image of the spinal cord. Contrast pulse sequencing (mechanical index 0.25) was employed to evaluate its perfusion pattern. Two milliliters (2.0 mL) SonoVue^®^ was administered as an intravenous bolus injection through a three-way 20-gauge catheter into an auricular vein; this was immediately followed by an injection of 10 mL of normal saline solution. All imaging sequences were digitally recorded on cine clips for later analysis.

### Analysis of CEUS clips

Using the autotracking contrast quantification (ACQ) software (Siemens, Mountain View, Calif, USA), quantitative analysis was done offline. After review of the clip, four regions of interest (ROIs) were set by software on the epicenter of contusion sites, adjacent region rostral, adjacent region caudal and distant region. Parameters, including arrival time (AT), time to peak (TTP) and peak intensity (PI) of all ROIs, were acquired automatically by ACQ software.

### Assessments of neurologic function and histology

Neurologic and histological examinations were employed for assessments in neurologic function and tissue damage respectively. Neurological function was evaluated with two methods: 9 points original behavioral scoring test (from 1 to 9) and 7 points cage-climbing test (from 0 to 6) as Iwanami et al. described [8]. The higher score the animal was given, the better neurologic function it had. Briefly, original behavioral scoring test was used to assess its ability to perform basic motions. The cage-climbing test was employed to evaluate coordination of fore- and hindlimbs. Animals were sacrificed after the last neurological evaluation and then spinal cord specimens were processed for H&E stain. The percentage of necrosis area was measured by using Adobe Photoshop CS3 (Adobe, San Jose, CA) on three consecutive sections.

### Statistical analysis

One-way analysis of variance (ANOVA) was performed in comparison of AT, TTP, PI and neurological score after contusion. Two-tailed unpaired Student's *t* test was used for comparison of the diameter of vessel before and after injury. A *p* < 0.05 was considered to be statistically significant. Correlation between histology and neurological scoring on Day 14th after injury was performed.

## CONCLUSIONS

This present study verified that CEUS is a promising method for monitoring perfusion changes of spinal cord in overall views and real-time for a nonhuman primate model.
